# Transition metal-based nanoparticles as potential antimicrobial agents: recent advancements, mechanistic, challenges, and future prospects

**DOI:** 10.1186/s11671-023-03861-1

**Published:** 2023-06-07

**Authors:** Sonali Gautam, Dipak Kumar Das, Jasvinder Kaur, Anuj Kumar, Mohd Ubaidullah, Mudassir Hasan, Krishna Kumar Yadav, Ram K. Gupta

**Affiliations:** 1 Nano-Technology Research Laboratory, Department of Chemistry, GLA University, Uttar Pradesh, Mathura, 281406 India; 2 Department of Chemistry, School of Sciences, IFTM University, Moradabad, Uttar Pradesh 244102 India; 3 Department of Chemistry, College of Science, King Saud University, Riyadh, 11451 Kingdom of Saudi Arabia; 4 Department of Chemical Engineering, King Khalid University, Abha, Kingdom of Saudi Arabia; 5 Faculty of Science and Technology, Madhyanchal Professional University, Ratibad, Bhopal, 462044 India; 6 Environmental and Atmospheric Sciences Research Group, Scientific Research Center, Al-Ayen University, Thi-Qar, Nasiriyah, 64001 Iraq; 7 Department of Chemistry, Kansas Polymer Research Center, Pittsburg State University, Pittsburg, KS 66762 USA

**Keywords:** Metal, Nanomaterials, Antibiotic, Antimicrobial agents, Mechanisms, Structural parameters

## Abstract

Bacterial transmission is considered one of the potential risks for communicable diseases, requiring promising antibiotics. Traditional drugs possess a limited spectrum of effectiveness, and their frequent administration reduces effectiveness and develops resistivity. In such a situation, we are left with the option of developing novel antibiotics with higher efficiency. In this regard, nanoparticles (NPs) may play a pivotal role in managing such medical situations due to their distinct physiochemical characteristics and impressive biocompatibility. Metallic NPs are found to possess extraordinary antibacterial effects that are useful in vitro as well as in vivo as self-modified therapeutic agents. Due to their wide range of antibacterial efficacy, they have potential therapeutic applications via diverse antibacterial routes. NPs not only restrict the development of bacterial resistance, but they also broaden the scope of antibacterial action without binding the bacterial cell directly to a particular receptor with promising effectiveness against both Gram-positive and Gram-negative microbes. This review aimed at exploring the most relevant types of metal NPs employed as antimicrobial agents, particularly those based on Mn, Fe, Co, Cu, and Zn metals, and their antimicrobial mechanisms. Further, the challenges and future prospects of NPs in biological applications are also discussed.

## Introduction

From a historical perspective, infection due to bacteria has been considered a key factor in the life and death of human beings. Most common bacterial infections include “pneumonia, wound infections, bloodstream infections (sepsis), and sexually transmitted diseases like gonorrhea,” as well as being accountable for other major epidemics [1]. Microbes can be severely pathogenic, causing disease if they succeed in overpowering the immune system. Some microbes, which are so-called opportunistic pathogens, only cause disorder in the correct circumstances. Opportunistic pathogens do not typically cause an infection in healthy individuals; however, the risk of exposure increases with a poor immune system, which is vulnerable or impaired by, for instance, cancer chemotherapy, certain disorders (such as HIV/AIDS), or starvation [2].

Such pathogens frequently arise from the microbial population of the organism, including on the skin or in the gut. Numerous microbial strains, namely *Salmonella, Campylobacter*, and *E. coli*, can transmit and are infected by water and food. Bacteria are often spread through livestock, either directly or indirectly, to individuals and cause illness. These infections are known as zoonotic infections. Some pathogens, such as *N. gonorrhea* and *C. trachomatis*, are transmitted by sexual intercourse. In conjunction with better health and cleanliness through the use of preventive vaccines, and improved awareness of bacteria, the implementation of antibiotics for the treatment of infectious diseases has significantly reduced deaths from pathogenic bacteria. Nevertheless, antimicrobial resistance among microbes is now contemplated, leaving us with many common infectious diseases despite successful treatments. In certain places around the world, pathogenic strains are now common, and more and more individuals suffer from infectious diseases since antibiotics have stopped functioning [3, 4].

The discovery of antibiotics is considered one of the greatest medical achievements of the twentieth century [5]. But the challenge was the lack of effectiveness due to antibiotic resistance bacteria, which might be due to the ability of the microorganism to resist either by inactivating them or by reducing the therapeutic effectiveness of antibacterial agents. Non-judicious use and misuse of antibiotics significantly contribute to the antibiotic resistivity [6], leading to long time illness and even higher death rates [7]. Although very complex, antibiotic resistance may follow enzymatic pathway modified by β-lactamases, acetyltransferases, or aminoglycoside enzymes [8]. Despite the growing need for new antibiotics, global disincentives to their use have significantly reduced the volume of sales compared to other medicines (e.g., those used for chronic diseases) [9]. Therefore, ground-breaking and efficient antimicrobial agents become the need of the hour to address the above challenge.

In several areas, particularly infectious mechanisms, nanotechnology provides future possibilities [10, 11]. According to the Food and Drug Administration (FDA) and the International Union of Pure and Applied Chemistry (IUPAC), the term “nano” denotes any substance with characteristics or anomalies due to its size when those sizes are between 1 and 100 nm [12, 13]. The nanoparticles (NPs), in particular, have distinctive properties in contrast to their majority of chemical equivalents, for example, greater surface area and adaptability, which can improve their impact on a particular microbe and other infections [10, 11]. The benefit of such NPs preparations over traditional systems is that, via their specific targeting therapeutic effect, they could improve therapeutic efficacy and prevent complications. In this context, nanotechnology involves the use of chemotherapeutic drugs with NPs, drug delivery and diagnostic systems, or the application of NPs in biomedical devices [14, 15].

During the last decade, huge amounts of money have been accumulated in the international market for medicinal nanomaterials, with increasing interest in the field of biomedical applications. Although, based on various drug-resistant disease, the demand of novel antibiotics is increasing regularly, existing antibiotic cell volume is found to get reduced than other drugs due to global disincentives toward use of antibiotics. Unfortunately, the number of pharma companies used to get involved earlier in research to develop novel antibiotic has been reduced drastically to 50% compared to 1980. Such withdrawing companies rather engaged to develop drug for chronic diseases and specific area like cancer having higher market share. In order to control such trend and to bring back antibiotic using fascinating approach of nanotechnology research, an appealing reward policy toward introduction of every new antibiotic has been executed. Simulation of interest in pharma companies is created due to the large amount of incentives. With the renewed mission, pharma companies have invested a lot of revenue to research and develop new nanomaterials as promising antibacterial agents generally resistant to conventional antibiotics. Multiple varieties of metal nanoparticles are already used for vast range of industrial applications but after discovery of their antibacterial activity, metallic NPs started taking noteworthy to leading role in the market share since 2016. It has been predicted to exceed USD 3 billion dollar for NPs-based new antibiotics by 2024.

**Fig. 1:**
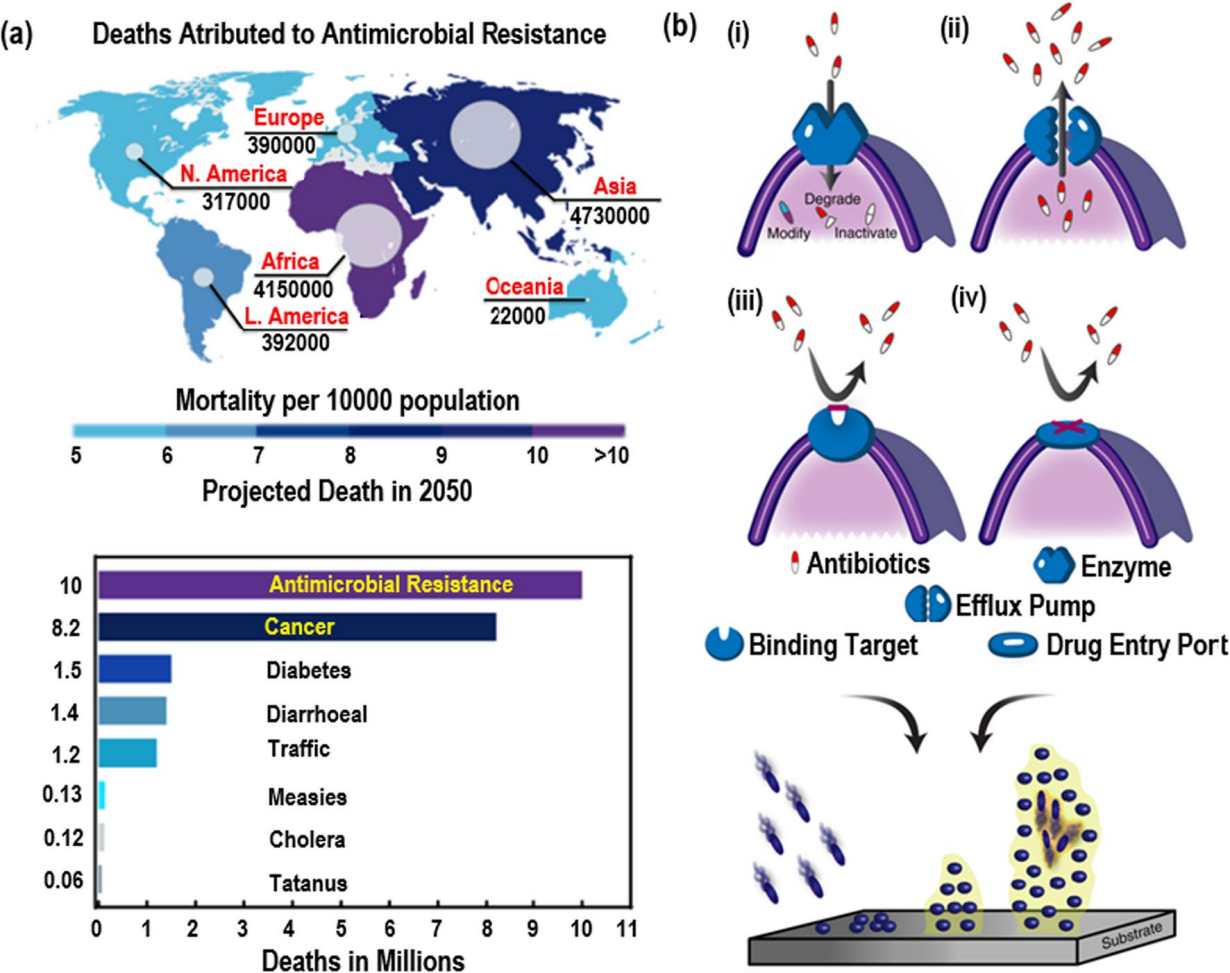
**a** Mortality projection of antimicrobial resistance over the global along with cancer and diabetes [16]. (Taken from online source; https://www.weforum.org/agenda/2016/09/antimicrobial-resistance-is-the-next-global-commons-issue/). **b** (i) modification, degradation, or inactivation of medication via enzyme production; (ii) alteration in cells efflux pump clearing the drug from the cells; (iii) alteration in the binding receptor to stop the arrival into the cell; (iv) blocking of medications entrance port to remove the influx into cells [17]. (This is an open-access article distributed under the terms of the Creative Commons Attribution (CC BY) license)

It is estimated that antibiotic resistance will reach epidemic levels globally by 2050, responsible for 10 million fatalities (Fig. 1a) [16]. Substantial deficiencies are observed in antibiotics, particularly poor antibacterial activities, a significant threat to beneficial microorganisms, and difficulty controlling and expanding work (Fig. 1b) [17]. Inside a biofilm, microbes encircle themselves within a polysaccharide and protein matrix, producing a slimy layer. Inhibiting antibiotic penetration through the film, this slimy matrix causes slower migration into the biofilm [18]. Until the antibiotic is able to disperse, this incompetent penetration triggers antibiotic discontinuation. Multicellular microorganisms are also capable of distinguishing a protective phenotype attributed to the anaerobic atmosphere. This contaminated environment prevents pathogens from developing biofilms and killing bacteria. Biofilms comprise enzymes that interrupt nutrients and acquire waste material, modify antibiotics, and antagonize antibacterial sensitivity in comparison with the anaerobic condition [19, 20].

There have been so many review papers published addressing advancements in various types of NPs for biological applications, but no one is focusing on particularly 3d-series transition metal-based NPs, their use as antimicrobial agents, or their mechanisms. This work will appeal to potential readers, including scientists, researchers, and academicians working in the realms of antimicrobial investigations using metallic nanoparticles, developing reactivity–functional relationships for NPs-based microbial agents, and investigating the mode of antimicrobial action of NPs. Further, various approaches have also been explored to determine the efficacy of the antimicrobial action of metal-based NPs. In addition, some basic antibacterial processes of NP that influence the various critical structures of microorganisms have also been discussed, such as oxidative stress induction, metal ion release, and non-oxidative harm. In the conclusion, the challenges and future prospects of NPs are also provided.

## Antimicrobial activity and mechanisms of metallic nanoparticles

Metallic NPs possessing significant antimicrobial properties may be remarkably helpful for eradicating sources of infection and diseases [21]. On the nanometer scale, the physicochemical and biological characteristics of NPs have been strengthened in terms of their surface area, distribution, and morphology. The antimicrobial properties of NPs, as suggested by earlier reports, depend on how they were synthesized [22]. Moreover, metal oxide NPs with unique chemical, magnetic, physical, biological, and optical properties are of great importance as antimicrobial agents for biologists. However, this field is only at its starting stage, and its mechanistic clarification is a problem that needs a detailed analysis. While the processes are not well known, research has shown that the delay/killing of bacterial growth is triggered by the interplay of one or more mechanisms and differs with metallic NP’s nature and chemistry. Reactive O-species (ROS) production is the primary form of antimicrobial action. In addition, during the production of ROS, cell membranes be damaged by electrostatic activity, metal ion homeostasis degradation, protein and enzyme dysfunction, genotoxicity, and protocoling [23], 24. Usually, bacteria have particular features that describe their metal-contact behavior with metal NPs, while the key toxicological effects of these NPs also work in bacteria in parallel. Both Gram-positive and Gram-negative bacteria bear negative charge domains on their cell walls [25]. This negative charge on cell walls is responsible for adopting metallic NPs via electrostatic forces or coordination-derived forces. Besides, metallic NPs also possess positive a charge on their surface, which can disrupt cell walls and boost NPs' permeability. In addition, metallic NPs can also produce extracellular metal ions that will reach the cell and disturb biological processes [26].

### Antimicrobial activity mechanisms of metallic nanoparticles

After the introduction of the use of NPs, it has become necessary to establish the mode of action of NPs, i.e., potential antibacterial mechanisms of NPs in medicine [27]. For instance, a change in the metabolic activities of bacteria has been reported due to metallic NPs [28], imparting added benefits to address diseases by eradicating bacteria. NPs can reduce the formation of biofilm on Ag-inhibited gene expressions [29]. Generally, to offer antibacterial property, NPs should be in contact with the bacterial cell via electrostatic, van der Waals, and hydrophobic [30] and receptor–ligand interactions, through which NPs have the liberty to enter the membrane and accumulate along with the metabolic route, thus affecting the functional and structural activities of the cell membrane. Afterward, NPs react with enzymes, ribosomes, DNA, and lysosomes, causing “oxidative stress, heterogeneous variations, improvements in the permeability of the cell membrane, electrolyte balance disorders, suppression of enzymes, deactivation of proteins, and alterations in the genetic expression as discussed below” [31].

#### Oxidative stress pathways

**Fig. 2:**
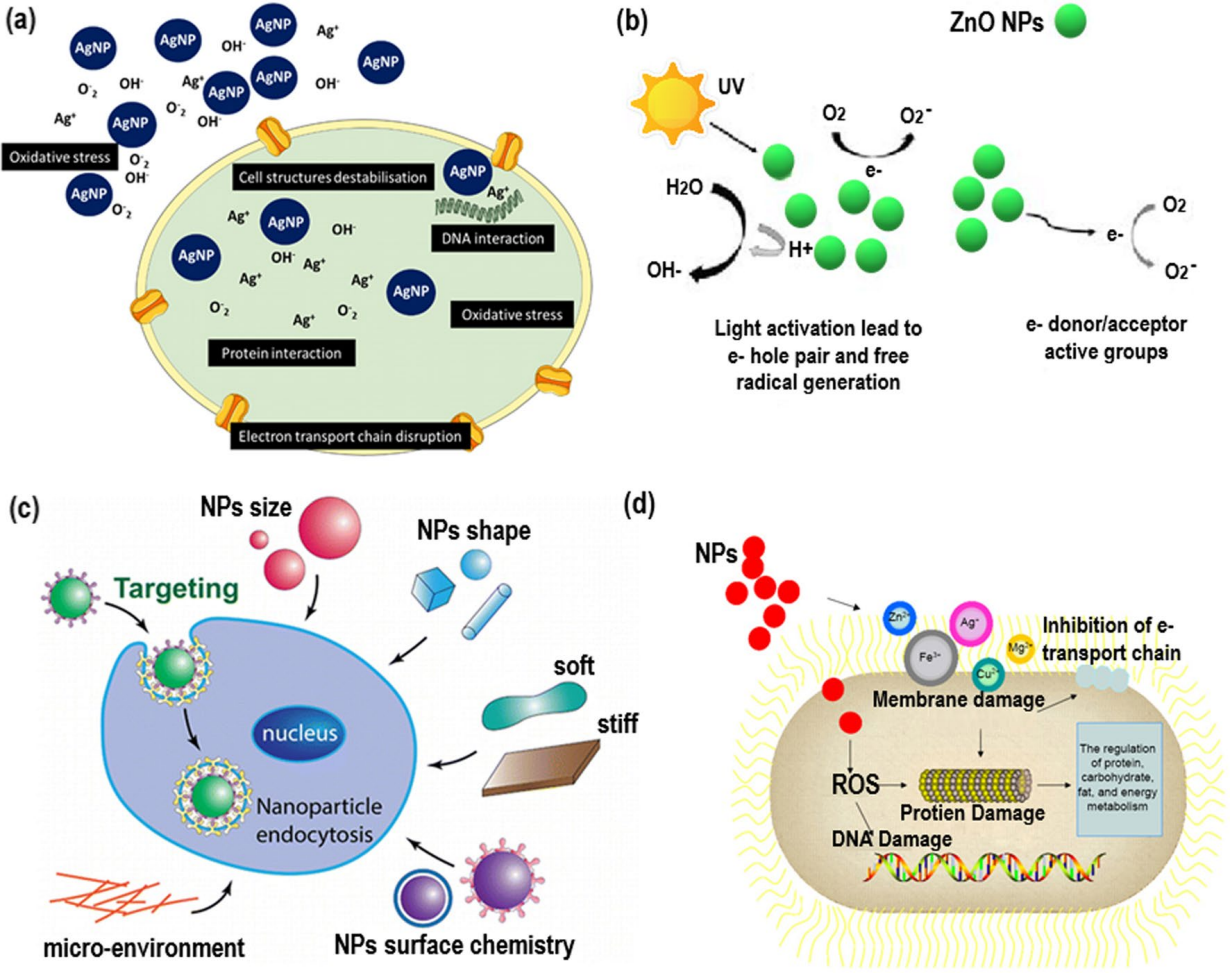
**a** Illustration of antimicrobial activity of Ag NPs mechanism [40]. (Licensed Creative Commons Attribution (CC BY) license). **b** ROS generation mechanisms on ZnO-NPs. Reproduced with permission [36]. (Licensed under a Creative Commons Attribution 4.0 International license, (CC BY 4.0)). **c** size, shape, and stiffness, etc., parameters of NPs affecting the biomechanical properties of cell membrane, as well as the local environment of the cells. Reproduced with permission [47]. Copyright 2015, American Chemical Society. **d** Illustration of antimicrobial activity of NPs mechanism. Reproduced with permission [10]. Copyright 2021, Dove Medical Press Limited

Different antimicrobial actions include causing damage to cell membranes by contacting them directly with metallic NPs, inhibiting the creation of biofilms, and producing free radicals as well as non-radical forms of reactive oxygen species (ROS). Under conditions of oxidative stress, the release of metal ions from NPs can result in the generation of reactive oxygen species (ROS). These ROS include peroxides (*O_2_^2−^), superoxide (*O_2_), hydroperoxyl (HO_2_*), hydroxyl radical (HO*), and singlet oxygen (^1^O_2_*). The production of ROS is the most significant of these effects. When reactive oxygen species (ROS) levels rise above the buffering capability of the cell, oxidative stress may develop. ROS have the potential to bring about lipid peroxidation, oxidative protein carbonylation, and deactivation of specific enzymes [32]. Moreover, several studies revealed that the permeability of the cell membrane was solely due to oxidative stress. Oxidative stress induced by reactive O-species (ROS) plays a significant role in the antibacterial mechanism of NPs. Restructuring, defect sites, and O-vacancies in crystals are the results of the creation of ROS via photocatalytic, electron–hole, and sonication-based strategies. ROS show different levels of dynamics and activity. For instance, CaO and MgO-based NPs can produce O^−2^, while ZnO-NPs produce H_2_O_2_ and OH^−^. However, CuO NPs can yield all these ROS. The O^−2^ and H_2_O_2_ species produce mild stress, which can be controlled by antioxidants like O^−2^-enzymes and catalase, whereas acute stress resulting in even death can be caused by OH and O_2_ as suggested by previous reports [33], 34. In the light of the photocatalytic effect, electrons in the valence band (VB) got stimulated and migrated to the conduction band (CB), resulting in the creation of corresponding holes in VB (H^+^) to produce ROS. In second strategy, TiO_2_ NPs in particular absorb light to create electron–hole pairs [35] which react with H_2_O in the presence of air on NPs surface to generate ROS (Fig. 2b) [36]. However, the third mechanism involves ultrasonic activation to produce ROS. These ROS further react with bacterial intracellular organic matter to show antimicrobial activity [37].

Besides, many authors suggested that adhesion of Ag NPs (which depend on size and their zeta potential) on bacterial surface might be the initial step involved in complicated bacterial inhibition mechanisms. Usually, smaller NPs can penetrate directly into the cell, whereas, larger NPs stay outside. In both situations, Ag NPs release Ag^+^ to bind with the cell membrane, de-stabilization of potential and causing H^+^ leakage, which enhances permeability [38]. After penetration, Ag NPs and Ag^+^ interact with structure and biomolecules like protein, lipid, and DNA inside the cell, causing malfunction. Further, Ag NPs are also responsible for creating ROS and other O-species. On the other hand, high levels of Ag^+^ generate high levels of oxidative stress. ROS may interact with respiratory chain proteins and inactivate the enzymes [39], and Ag^+^ can bind itself to DNA base pairs, damaging the H-bond between them (Fig. 2a) [40].

#### Dissolved metal ion pathways

In this approach, metal ions are believed to be released from metallic-oxide NPs and adsorb on cell membrane. Later on, they interact with functional groups (-COOH, -NH_2_ and –SH) of nucleic acids to deactivate the enzyme, causing the change in cell structure. However, in case of metal oxide suspensions, the weak antimicrobial activity is due to the fact that the metal ions have little impact on pH inside lipid vesicles [41]. Likewise, it is also reported that superparamagnetic Fe-oxides can penetrates inside the cell membrane, disturbing the transfer of electrons belonging to transmembrane. Moreover, heavy metal ions are reported to act as carriers of antimicrobial components indirectly [42].

#### Non-oxidative pathway

A non-oxidative pathway for antibacterial action of NPs was established by a study involving MgO NPs. The visibility of MgO NPs was not there in the cell during the rupture of bacterial cell membrane to make surface pores clearly visible, supporting the action of MgO to cause lipid peroxidation, thus indicating the inhibitory effect of these NPs to damage the cell membrane [43].

### NPs interaction with cell barrier

Usually, cell membrane components are responsible for the adsorption of NPs and Gram-positive/negative microbes. Lipopolysaccharides are the major constituents of Gram-negative bacterial cell walls and offer a negative charge density to attract NPs. However, NPs are also reported to have superior efficacy against Gram-positive bacteria in comparison with Gram-negative microbes. This fact could be attributed to the composition of cell wall of Gram-negative bacteria (lipopolysaccharides, lipoproteins, and phospholipids), which offers a secondary barrier that permits macromolecules inside. On the other hand, the cell walls of Gram-positive bacteria possess a porous, thin layer of teichoic acid to allow foreign molecules inside, resulting in damage and killing of cells [44]. Hyldgaard et al. [45] described that phospholipid of lipopolysaccharide membranes in *E. coli* cooperates with ε-poly-L-lysine to damage the cell membrane due to electrostatic attraction. In another report, antibacterial evaluation of nano-diamonds having various O-functionalities was performed and found that these nano-diamonds can connect covalently with –COOH/-NH_2_ functional groups on nearby proteins on cell walls. These works justified the idea that the bacterial structure can also influence the antimicrobial activity of NPs [46].

#### Expression of metabolic genes with NPs

The metabolic pathway in bacteria is an integrated, complex activity of living cells. Therefore, a change in metabolic activity can purposefully regulate the pathogenicity of bacterial cells. For instance, MgO and CuO NPs have been reported to change the expression of several proteins associated with bacterial N-metabolism, preventing the activity of nitrate and nitrite reductases considerably [39].

### Influencing factors of metallic nanoparticles' antimicrobial activity

The size, surface area, energy, charge and morphology, zeta potential, and crystal structure of NPs, including environmental parameters like bacterial strain and exposure time, can significantly affect the antimicrobial action of NPs (Fig. 2c) [47, 48].

#### Size

NPs of smaller size possess a larger surface area, resulting in a higher probability of penetration through the cell membrane [47]. In contrast, when Mg(OH)_2_ NPs of different sizes were analyzed, the smaller Mg(OH)_2_ NPs exhibited the least antibacterial activity, indicating that the size of NPs is not at all a conclusive parameter [49]. Shape: It is reported that various shapes of NPs cause different degrees of bacterial cell damage. For instance, a comparative investigation using pyramidal, spherical, and plate-type ZnO-NPs suggested that shape-specific ZnO-NPs combined with β-galactosidase exhibited their photocatalytic action via different pathways [50]. Roughness: Few reports suggest that size and surface area-to-mass ratio can endorse bacteriological proteins, followed by a decline in bacterial adhesion with the increment of roughness of NPs [51]. Doping modification; hetero-doping in NPs not only reduces the possibility of aggregation but also controls and regulates the interaction of NPs and bacteria effectively. For instance, Au doping in ZnO-NPs was believed to be the reason for the enhancement in photocatalytic activity of ZnO-NPs, increasing the ROS on the ZnO-NPs surface. Moreover, Au doping in ZnO-NPs improved the light absorption, altering the band gap of ZnO-NPs [35].

#### Zeta potential

Recent reports have suggested that the zeta potential has a significant impact on bacterial adhesion. The negatively charged cell membrane opens the possibility for positively charged NPs to get adsorbed on its surface via electrostatic attraction forces. In addition, the probability of NPs getting accumulated in the infected area enhances the vascular permeability of NPs, exhibiting good antimicrobial activity. Moreover, positively charged domains were found to be responsible for the production of ROS as compared to negatively charged and neutral NPs [52].

#### Environmental conditions

Several investigations have suggested that different environmental situations like temperature and pH can cause significant variation in the antimicrobial potency of NPs, due to fluctuations in the extent of ROS. Besides, a decrease in pH can increase the dissolution of ZnO-NPs, thereby enhancing their antimicrobial activity. In addition, lower pH causes the surface of NPs to become positively charged, which is favorable for the negative charge group of bacterial cell barriers. The pH and osmotic pressure can also affect the agglomeration, surface charge, and solubility of NPs, altering their antimicrobial activity (Fig. 2d) [10].

### Antimicrobial resistance mechanisms of metallic nanoparticles

Improper and non-prescribed (by authorized practitioners) use of antibiotics, particularly in developing countries, has created the possibility of bacterial resistance to antibiotics. In addition, the inappropriate selection of antibiotics has added a problem to the above crisis. Based on the source of resistant genes, antibiotic resistance can be classified into: (i) intrinsic resistance, which can result from the natural mutation of exogenous genes; and (ii) acquired resistance, due to the attainment of resistance genes from other organisms [53], 54.

**Fig. 3:**
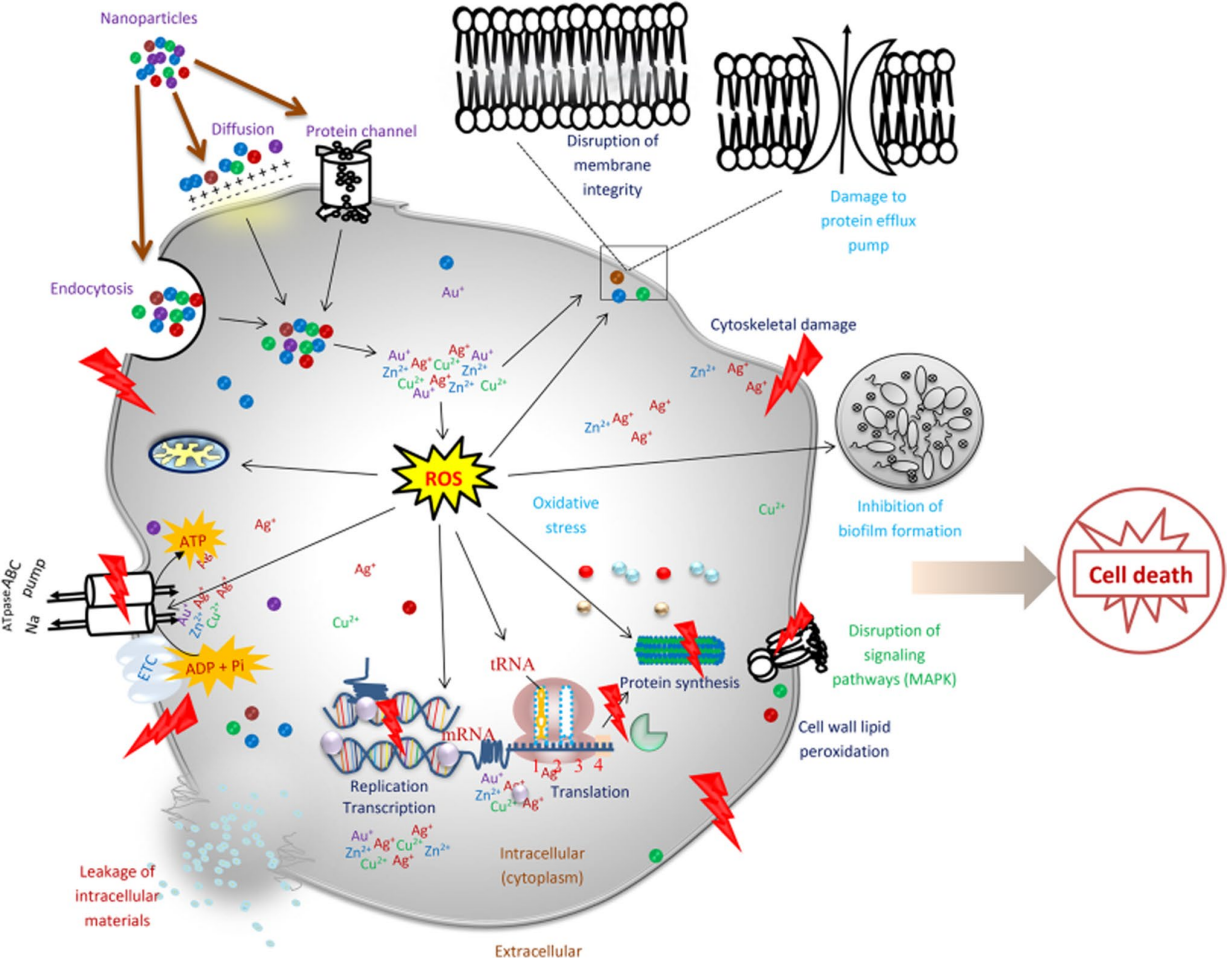
Mechanism of nanoparticles activity against bacteria. Taken with permission [55]. Copyrights @2021. Springer Nature publishing group

Certain resistance mechanisms are proposed due to the alteration of proteins as well as particular enzymes. The principle mechanisms are associated with target changes, the creation of passive enzymes, the application of efflux pumps, the appearance of hindrances to antibiotic infusion, biofilm formation, etc. Before the concept of NPs appeared, three different approaches to addressing antibiotic resistance were in practice, which included the formulation of novel drugs, the use of higher antibiotic doses, and the protocol of using many antibiotics [56, 57]. Nevertheless, the formulation of a novel drug cannot be designed immediately with the mutation of bacteria, and antibiotics at higher doses may be associated with toxicity problems beyond the tolerance level. Moreover, these protocols will increase antibiotic misuse and the possibility of multidrug-resistant strains [58]. On the other hand, when NPs are conjugated or coated with other compounds, their activities are considerably improved. In fact, mixing NPs with antibiotics can help lessen the prevalence of drug-resistant microorganisms. Antibiotics and NPs with varying modes of action make resistant microbes more susceptible. If a microbe is resistant to one type of antibiotic, a second type of antimicrobial could be able to destroy it. Antibiotics can be transported in NPs, making it easier for them to penetrate bacterial cell walls. The antibiotic weakens the cell wall, which lets the NPs and their complicated contents inside [55]. A systematic mechanism of NPs activity against bacteria and the way of resistance is shown in Fig. 3.

## Recent developments in antimicrobial agents based on metallic nanoparticles

Usually, drug resistance is one of the negative effects on public health that can be caused by the routine, non-scientific consumption of antibiotics [59]. That is why it is so important to find new, effective bactericidal components to combat the pervasive problem of antibiotic resistance. Because of their preventive role against microbial treatment resistance, NPs have been proposed as a possible alternative to antibiotics. In this section, we reviewed the recent advancements on 3d-series transition metal (Mn, Fe, Co, Cu, and Zn)-based NPs for the antimicrobial applications [60–62].

### Mn-based nanoparticles

Khan et al. [63]*,* synthesized MnO nanoparticles using *A. indicium*, then evaluated green synthesized AI-MnO-NPs, which displayed higher and comparable antibacterial efficacy toward *B. subtilis* and *S. aureus* to the antibiotic drug, while the antibacterial effect was observed with the plant extract. Several properties, like semipermeability and the oxidative phosphorylation process, are affected by the structure and composition of cell the membrane. Thus, lipid peroxidation is harmful to various forms of life. Moreover, the typical functions associated with an intact membrane, such as respiratory activity, are destroyed by the lipid peroxidation (LPR) [64]. Mechanisms in membrane structure are originated by LPR cause modifications in membrane-bound proteins, electron mediators, and alignment of composites across the cell membrane, seepage of K^+^ ions, and succeeding functional changes, thus leading to cell death on interaction with Mn_0.5_Zn_0.5_Fe_2_O_4_NPs [65, 66]. Further, this research group demonstrated that Mn-doped ZnO-NPs displayed exceptional antimicrobial efficacy in comparison with ZnO-NPs. This fact was attributed to the large surface area and smaller particle size of Mn-doped NPs. Thus, the synthesized nanoparticles hold enormous potential for use in the cosmetic, nutraceutical, and pharmaceutical industries [67].

Muhamed et al. [68]*,* synthesized manganese oxide NPs using lemon extract and curcumin extract. The NPs were characterized by UV–Vis and FT-IR techniques, which indicated the development of functionalized MnO-NPs. As indicated by SEM, the NPs were reported to have a spherical with a size of about 50 $\pm$ 5 nm. The NPs were then evaluated for their antimicrobial activity against *S. aureus*, *B. subtitles*, *S. typhus*, *C. albicans*, *C. lunate*, and *T. simii* pathogens. Consequently, they observed that curcumin–aniline modified MnO-NPs are superior in antibacterial efficacy by preventing bacterial growth. The outcomes of this study may offer possible discoveries in the realms of antimicrobial agents and biomedical devices [69] [70]. Azhir et al. synthesized Mn_3_O_4_ NPs by using the precipitation method. Having 10–30 nm average particle size, these NPs possessed antimicrobial potency against bacterial pathogenicity like *E.coli* and *S. aureus*. The qualitative analysis of antibacterial assessments indicated that *E. coli* were more sensitive to these NPs than Gram-positive bacteria (*S. aureus*) [71]. Further, Joshi et al. synthesized manganese dioxide NPs showing their effective antimicrobial activity against *S. aureus*, *P. vulgaris*, *S. typhi*, *S. mutants*, and *E. coli* and MDO NPs [72]. Kumar et al. also prepared Mn_3_O_4_ NPs at various pH levels that indicated the regulation of pH is very important to control particle size of synthesized materials. These NPs were assessed for their antimicrobial efficacy against both Gram-positive and Gram-negative microbes by employing disk diffusion approach, demonstrating their greater antibacterial action against Gram-negative bacteria [73].

**Fig. 4:**
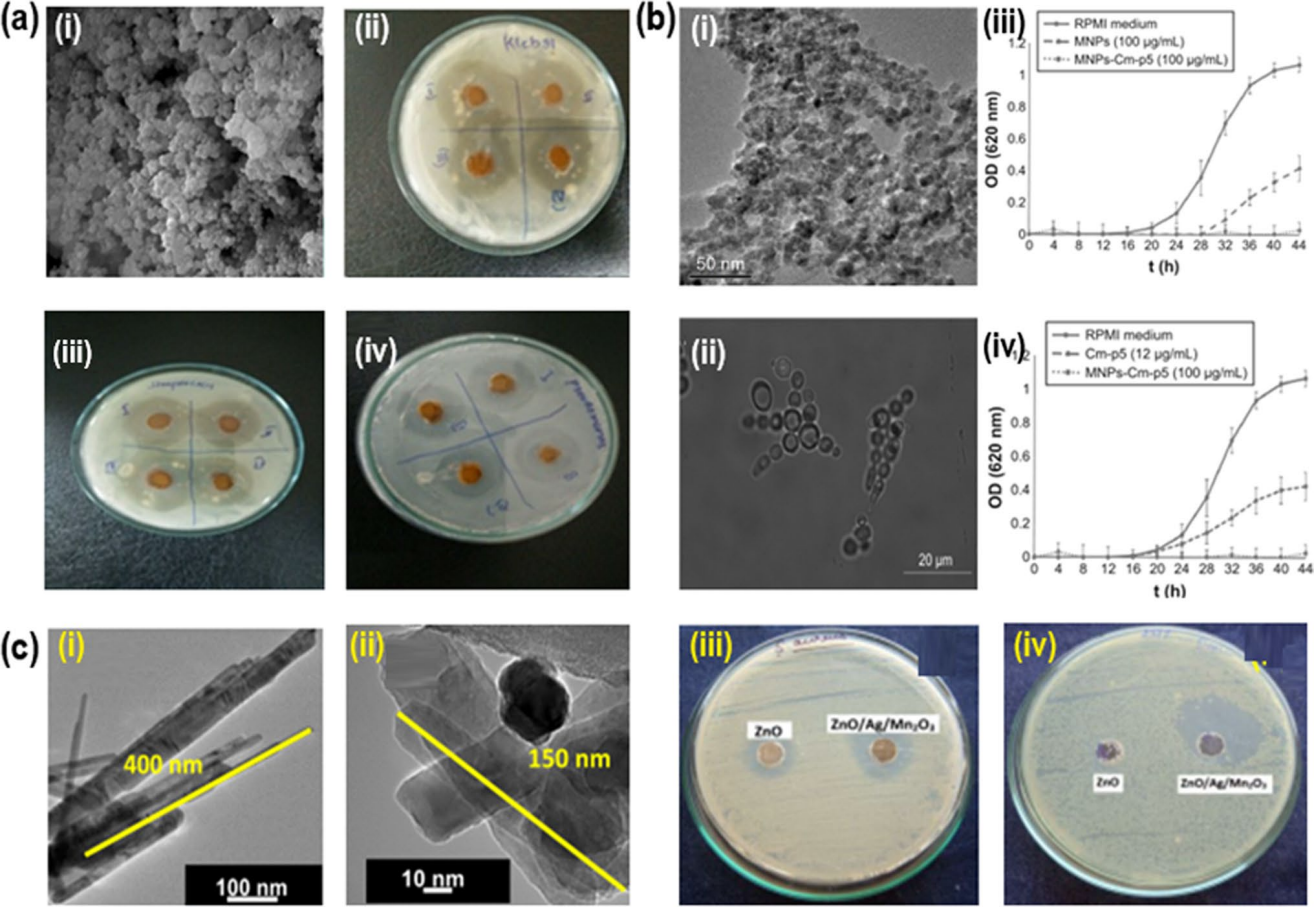
**a** (i) SEM image of Mn-dioxide NPs, and their antimicrobial activity against (ii) *K. pneumonia*, (iii) *S. aureus*, (iv) *P. aeruginosa* [75] (This article is an open-access article distributed under the terms and conditions of the Creative Commons Attribution (CC BY) license). **b** (i) TEM image of MnFe_2_O_4_-NPs, (ii) TEM image of MnFe_2_O_4_-NPs along with *C. albicans*, (iii) *C. albicans* growth curves for cm-p5-conjugated MnFe_2_O_4_-NPs and modified MnFe_2_O_4_-NPs in RPMI-1640 medium. (iv) *C. albicans* growth curves of cm-p5 peptide and cm-p5-conjugated MnFe_2_O_4_-NPs in rPMI-1640 medium [76]. (This article is an open-access article distributed under the terms and conditions of the Creative Commons Attribution (CC BY) license). **c.** TEM images of (i) pure ZnO, (ii) ZnO/Ag/Mn_2_O_3_, and pictures of zone of inhibition by pure ZnO and ternary ZnO/Ag/Mn_2_O_3_ against (iii) *S. aureus*, and (iv) *E. coli*. Reproduced with permission [77]. Copyright 2021 RSC Publication Group

Besides, graphene incorporated NPs were also recognized as potential nanocomposites with antimicrobial activity. For instance, Lara et al. synthesized manganese ferrite NPs incorporated with graphene (MnFe_2_O_4_-G) and characterized them using various analytical techniques, indicating MnFe_2_O_4_ NPs of 25 nm size dispersion on graphene sheets. The MnFe_2_O_4_ NPs and MnFe_2_O_4_-G nanocomposite (NC) were evaluated for their antimicrobial efficacy against *E. coli*, and the highest potency was observed for the nanocomposite, due to the synergetic effect of graphene and MnFe_2_O_4_ NPs [74]. Cherian et al*.* [75]*,* prepared the spherical MnO-NPs (Fig. 4a(i)), demonstrating their high antimicrobial activity (Fig. 4a(ii–iv)). Lopez et al. reported the citric acid-coated MnFe_2_O_4_-NPs with significant antifungal activity (Fig. 4b(i–iv)) [76].

In addition, interesting work was conducted to estimate the antimicrobial potency of ZnO/Ag/Mn_2_O_3_ NC and compared it with ZnO, Ag, and Mn_2_O_3_ NPs, indicating that synergic effect in ZnO/Ag/Mn_2_O_3_ NC was accountable for the improved antimicrobial activity (Fig. 4c(i–iv)). This work is more important from the point of view of utilizing the concepts of synergistic effect to design various materials not only for biological purposes but also for energy and catalytic sectors [77].

### Fe-based nanoparticles

Due to the better biocompatibility of Fe atoms, Fe-oxide-based NPs have been used as effective materials for therapies and other biological applications [78]. Santoshi et al. synthesized FeO NPs using *desmodium gangeticum* (DG) root aqueous extract and characterized them by multiple techniques, indicating their spherical shape with a 25–35 nm size and less aggregation. The synthesized FeO NPs were tested for antibacterial activity against *E. coli*, *B. subtilis*, and *S. aureus* using the agar-well diffusion method. Antimicrobial function of DG extracts, however, there were substantially high nanoparticles. In addition, it was suggested that bacteria were required to absorb Fe^3+^ and reduce Fe^3+^ to Fe^2+^ species. The Fe ion is in direct proportion to the iron's bacteriostatic impact on hydroxyl unrestricted inhibition concentration and mediates radical formation [79]. Arias et al*.* suggested that the ability for microbial toxicity of magnetic NPs and metal ion release affected Celtic homeostasis and coordination of proteins, including membrane depolarization with consequent cell integrity deficiency [80].

Dalia et al. synthesized two samples of iron oxide NPs by utilizing brown (*C. sinuosa*) and red (*P. capillacea*) seaweed extracts. These Fe_3_O_4_ NPs demonstrated a wide variety of antibacterial potential against the evolution of Gram-negative bacteria (8.37 $\pm 6.62 \mathrm{mm}$) and Gram-positive bacteria (5.75 $\pm 2.25 \mathrm{mm}$). Furthermore, Fe_3_O_4_NPs from C. *sinuosa* displayed exceptional antifungal effectiveness against *Aspergillus flavus* (9 mm) and *F. oxysporm* (6 mm) in comparison with Fe_3_O_4_ NPs from *P. capillacea* (7 and 5 mm) and against control [81]. Yosmery et al. synthesized iron NPs using leaf extracts of *E. robusta* and evaluated their antimicrobial activity against different pathogenic microorganisms such as *P. aeruginosa*, *E. coli*, *S. aureus*, and *B. subtilis*, indicating their high antibacterial activity [82]. Further, Ansari et al. also found similar results for iron oxide NPs against these bacteria [83].

Marimuthu et al. synthesized iron oxide NPs by the co-precipitation method using CoCl_2_, MnCl_2_, FeCl_3_ mixtures in NaOH solution, and their antibacterial potency was investigated against *B. subtillis* and *E. coli*. The results showed that these NPs had mild antibacterial properties [84]. Further, Avval et al. synthesized Fe_3_O_4_ NPs using *K. alvarezii* plant extract with the combustion method. The antimicrobial activity of these NPs was assessed against *S. aureus* and *E. coli* and also assessed through impedance techniques. The findings showed that, relative to traditional approaches, the synthesis of Fe_3_O_4_ NPs was economically cheap, and the nanoparticles displayed a greater potential in the elimination of harmful textile dyes in an environmentally friendly manner [85]. Further, Saqib et al. synthesized iron oxide NPs using the process of co-precipitation, and a spinal shaped morphology was attributed to these NPs, indicating their promising antimicrobial efficacy against *S. aureus* and *E. coli.* In this analysis, the composition of the synthesized NPs was investigated in order to protect them against harmful bacteria [86].

**Fig. 5:**
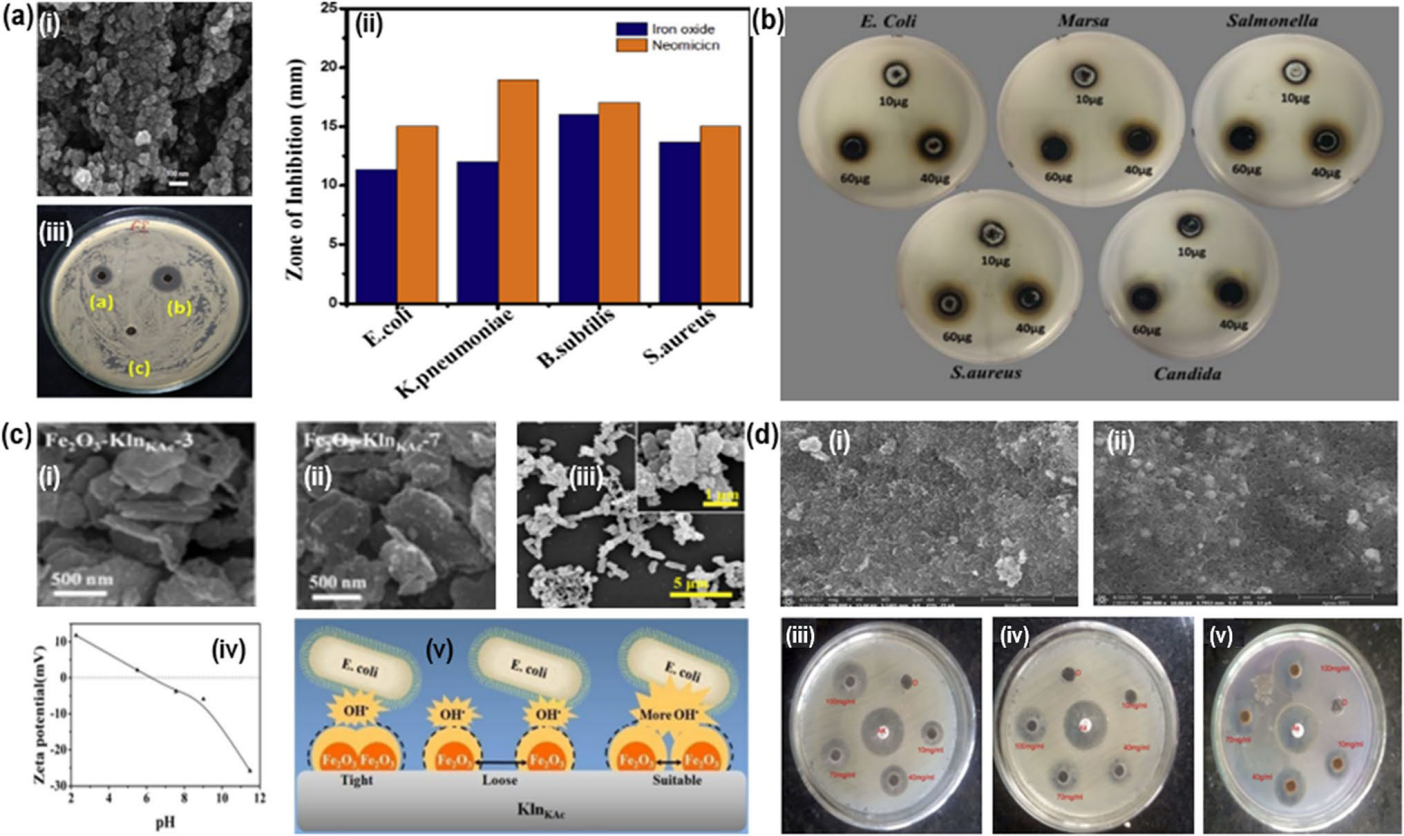
**a** (i) SEM image of *S. cordifolia* mediated α-Fe_2_O_3_ NPs, (ii) comparison of Zone of inhibition for green synthesis of *S. cordifolia* mediated α-Fe_2_O_3_ NPs and standard antibiotic with 50 μ g/ml against tested bacteria, (iii) Zone of inhibition of α -Fe_2_O_3_ (50 μ g/mL). Reproduced with permission [87]. Copyright 2021 Elsevier Publication Group. **b** Ability of Fe-NPs to inhibit bacterial growth [88]. (This article is an open-access article distributed under the terms and conditions of the Creative Commons Attribution (CC BY) license). **c.** (i & ii) SEM images of Fe_2_O_3_, (iii) SEM images of *E. coli* after incubation with Fe_2_O_3_, (iv) Zeta potential at different pH for Fe_2_O_3_, (v) Schematic illustration of antibacterial activity of Fe_2_O_3_ composites. Reproduced with permission [89]. Copyright 2021 RSC Publication Group. **d.** SEM pictures of (i) Fe-oxide NPs, and (ii) Himalayan honey loaded Fe-oxide NPs. Activity zone of inhibition displayed by (iii) Himalayan honey (iv), Fe-oxide NPs, and (v) Himalayan honey loaded Fe-oxide NPs against both Gram-positive/negative bacteria. Reproduced with permission [90]. Copyrights 2019, Taylor & Francis publishing group

Naga et al*.* fabricated α-Fe_2_O_3_ NPs employing *S. cordifolia* plant extract and further evaluated their antibacterial activity (Fig. 5a(i)). These *S. cordifolia* mediated α-Fe_2_O_3_ NPs showed potential antibacterial efficacy against different Gram-positive and Gram-negative microbes (Fig. 5a(ii–iii) [87]. Further, Dana et al. synthesized Fe-NPs by a phyto-assisted method employing *Acacia nilotica* seedless pod extract and characterized using multiple techniques [88]. These synthesized Fe-NPs were found to be potential agents to dismiss the pathogenic effect of a few human opportunistic microbes fits to Gram-negative/positive bacteria (Fig. 5b). By adjusting the solution pH and adding the kaolinite nanosheets (Fe_2_O_3_-Kln KAc), Long et al. [89] claimed that they were able to increase the antibacterial efficacy of Fe_2_O_3_ NPs by monitoring the distribution density over their surface. The results showed that by carefully controlling the distribution density across the Fe_2_O_3_NPs, the OH– level could be adjusted, significantly improving the NPs' antibacterial efficacy (Fig. 5c(i–v)). The biological activity of Fe-oxide NPs was shown to be significantly enhanced after Himalayan honey loading on their surface, as reported by Neupane et al. As a result, Himalayan honey loaded Fe-oxide NPs showed promise as a safe and effective replacement for conventional antioxidant and antibacterial agents (Fig. 5d(i–v)) [90].

### Co-based nanoparticles

Arsalan et al. [91]*,* prepared Co_3_O_4_NPs and controlled their size to under 100 nm. The results of antimicrobial studies have shown a remarkable impact of Co_3_O_4_ NPs on *S. aureus, E. coli*, in terms of more changes in the outer membrane of Gram-positive bacteria in the form of Co-ions. Rashmi et al. [92]*.* reported a plausible mechanism of antimicrobial action of prepared CoFe_2_O_4_ NPs. Such results indicated the possibility of killing both Gram-positive and Gram-negative cells, as both are negatively charged cells that favor or release ions from electrostatic contact with NPs. Therefore, the antimicrobial mechanism of ferrites is involved in the development of free radicals, primarily ROS. In addition, it is hypothesized that the oxidative stress on bacteria could be caused by the release of cytoplasmic materials (sugar and protein), and the breakage of the cell membrane contributes to the lack of mitochondrial activity, resulting in the death of cells [93]. However, Gram-negative bacteria are more susceptible to CoFe_2_O_4_ NPs, the reason may be due to the fact that Gram-negative bacteria vary in their structural and chemical constituents that are surrounded by lipopolysaccharide outer layers, whereas peptidoglycan is less rigid and can readily break [94]. Nonetheless, the exact procedure behind antimicrobial behavior is not known for CoFe_2_O_4_ NPs. The results indicated that the CoFe_2_O_4_ NPs intracellular fluid leakage from all tested samples may be due to bacteria [95].

Raza et al. synthesized Co-oxide NPs and evaluated their antimicrobial efficacy against *E. coli, P. aeruginosa* and *B. subtilis* at various concentrations. The antibacterial test showed particles at higher concentrations exhibited better antibacterial performance [96]. Hedaiat et al. synthesized similar NPs using the Taguchi method and found that these NPs can be used in the manufacturing of dental and medical devices with antibacterial efficacy due to their optimum antibacterial properties [58]. Further, Safaei et al. prepared Co-oxide NPs (Co_3_O_4_ NPs) using leaf extracts of populous ciliates [97]. Their antimicrobial activity was investigated against *B. lichenifermia, K. pneumonia, B. subtillus*, and *E. coli,* and it was found that by increasing the concentration of Co-oxide NPs, antibacterial activity was increased [98], whereas Anwar et al.* reported* three types of Co-based NPs (Co_3_O_4_ nanograins, Co_3_(PO_4_)_2_ micro-flakes and Co(OH)_2_ nanoflakes). The amoebicidal, encystation, excystation, and host cell cytopathogenic efficacy studies were performed to investigate the anti-acanthamoebic effects of Co-NPs for the development of antiamoebic nanomedicine [99, 100]. Kirupagaran et al. synthesized Co-NPs of spherical shape with 54–125 nm average diameter using aqueous and methanol extracts of *morusindica* leaves. These Co-NPs were synthesized using the green route and found to be potential antibacterial agents [101].

**Fig. 6:**
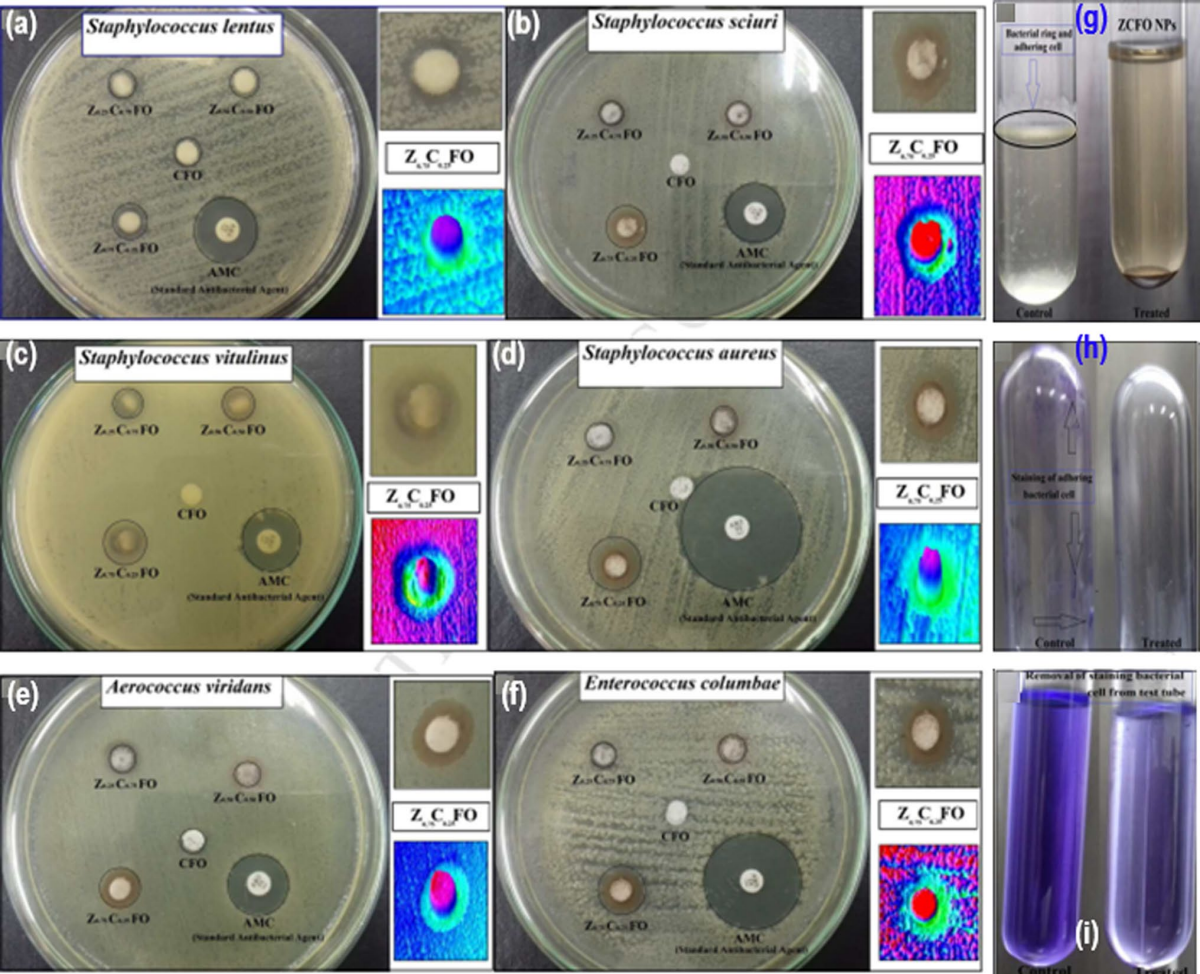
Antibacterial activity of CFO, Zn_x_Co_1-x_Fe_2_O_4_ NPs; x = 0.25, 0.50, and 0.75 against **a** *S. lentus*, **b** *S. sciuri*, **c** *S. vitulinus*, **d** *S. aureus*, **e** *A. viridians*, and **f** *E. columbae* as ZOI (mm). Antibiofilm activity of Zn_0.75_Co_0.25_Fe_2_O_4_ NPs against *E. columbae* where **g** Test method for biofilm detection in the presence and absence of ZCFO NPs, **h** Staining of the adherent bacterial cell using crystal violet stain, and **i** Crystal violet decolorization by ethanol for quantitative analysis. Reproduced with permission [102]. Copyright 2021 Elsevier Publication Group

Maksoud et al. reported Mn_0.75_Co_0.25_Fe_2_O_4_ mixed NC and characterization using various analytical techniques. The outcomes of antibacterial studies exhibited that the most efficient composition of NCs was Zn_0.75_Co_0.25_Fe_2_O_4_ (20.0 ppm) that displayed the best activity against *S. aureus*, *E. columbae*, and *A. viridians* (Fig. 6(a–i)) [102].

### Cu-based nanoparticles

Azam et al. prepared chitosan-Cu-based NPs using a chemical process and characterized by various techniques indicating their size in the range of 200–350 nm. The antimicrobial investigation was carried out against *S. choleraesuis*, *S. aureus, P. aeruginosa,* and *B. subtilis.* The results indicated their potential applications in pharmaceutical and biomedical sciences [103]. Manjari et al. fabricated Cu-oxide NPs using *A. agnoidea* flower extract and the TEM images showed their sizes in the range of 20–45 nm. These Cu-oxide NPs were found to be potential antimicrobial agents against various bacteria [104]. Further, Harish et al. reported CuO to have antimicrobial activity against *K. pneumonia, S. typhimurium*, and *E. aerogenes*. The minimum inhibitory concentration for these three Gram-negative bacterial strains, viz. *K. pneumonia, S. typhimurium,* and *E. aerogenes*, was observed to be 0.55, 0.15, and 0.30 µg/mL, respectively [23].

Linlin et al. proposed interesting results, indicating that NPs can attack bacterial cells by several mechanisms, developing reactive O-species that lead to disruption of the membrane. In this case, direct contact with the cell membrane occurs as certain metal-based NPs can produce metal ions, for instance, in electron transport chain inhibition and bacterial metabolic control processes [10]. Maqusood et al. synthesized copper oxide nanoparticles by precipitation and characterized with multiple techniques such as XRD, FESEM, EDS, and HRTEM. The average size of particles indicated by TEM and XRD was about 23 nm. CuO NPs have demonstrated outstanding antimicrobial activity against different bacterial strains. Furthermore, *E. coli* and *E. faecalis* demonstrated high sensitivity to CuO NPs, while *K. pneumonia* [105].

Hemalatha et al. synthesized CuO nanoparticles using *Eichhorni acrassipes* leaf extract. CuO NPs characterized with techniques like XRD, FT-IR, UV–Vis, SEM, and EDX. The size of NPs is 20–25 nm, and the CuO NPs show excellent antibacterial activity against *S. pneumonia, S. aureus* and *K. pneumonia* [106]. Amiri et al. synthesized copper oxide nanoparticles. Nano-copper oxide used in this study showed good antimicrobial efficacy against the investigated dental caries pathogens whereas a low efficacy for three species of candida. Thus, this NP can be introduced as a potential controlling agent in the prevention of dental caries or other infections [107, 108]. Asamoah et al*.* synthesized Cu-oxide and Zn oxide nanoparticles using the wet chemical reduction method. Prepared NPs were investigated by TEM, XRD, FT-IR, UV–Vis. The size of Zn oxide nanoparticles was observed to be ~ 15 nm and spherical in shape, whereas CuO NPs were assumed to have a nanorod-like shape. The two oxide NPs were studied for their antimicrobial potency against *S. aureus* and *E. coli*. The findings revealed that CuO had better antibacterial activity relative to ZnO [109].

**Fig. 7:**
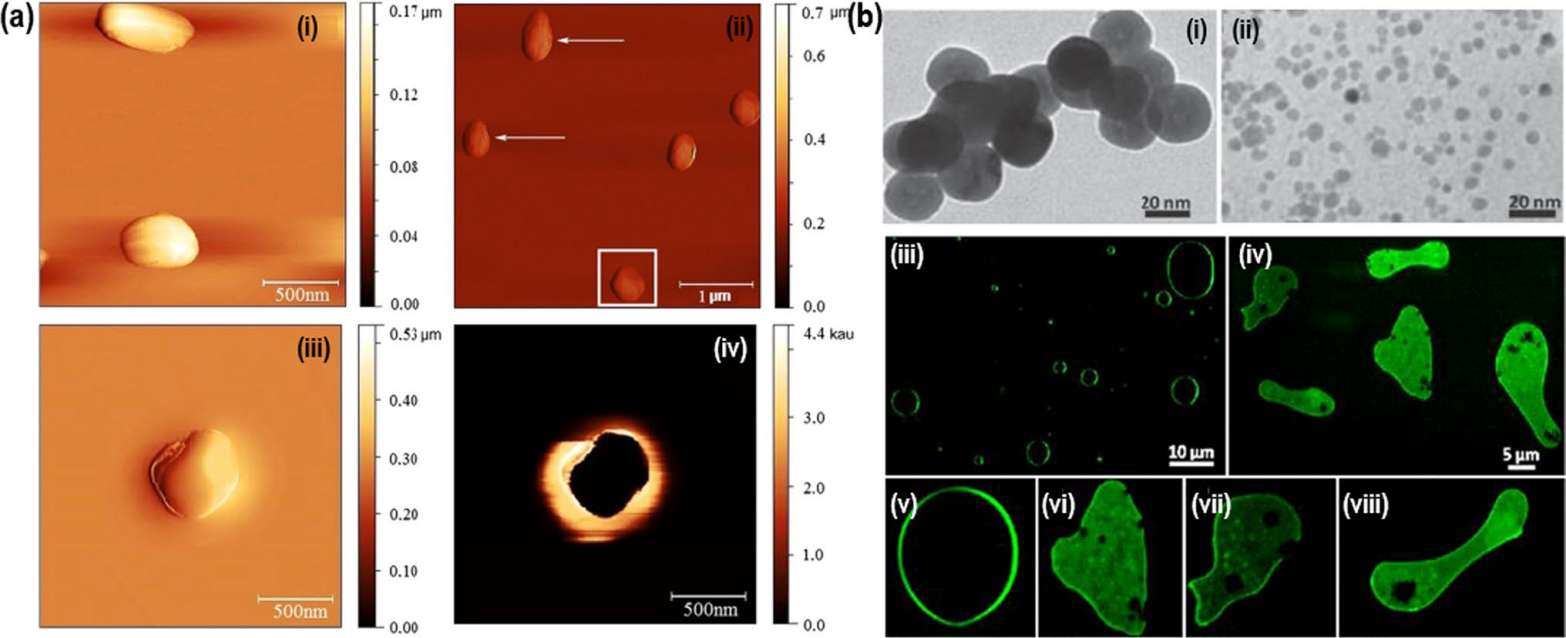
**a** Topography pictures of untreated (i) and treated (different magnifications: ii, (iii) with treated *C. albicans* (iv). Reproduced with permission [110]. Copyright 2021 Elsevier Publication Group. **b** (i–ii) TEM pictures of Cu@Cu_2_O NPs, and optical fluorescence microscopy images of S. cerevisiae cells (iii) in absence of NPs/NCs (control) or (iv) in the presence of NCs and pictures of (v) control cells and (vi & viii) cells treated with NCs after their exposure to 30 Hz magnetic field. Reproduced with permission [111]. Copyright 2021 Elsevier Publication Group

Bogdanović et al. [110] prepared Cu-hydrosol-based NPs with a narrow size distribution and applied them to study antimicrobial potency against *E. coli, S. aureus* and *C. albicans*. Further, the morphology of strains exposed to the Cu-NPs was investigated by employing atomic force microscopy (Fig. 7a(i–iii)). The antifungal efficacy of Cu_2_O NPs was investigated for these NPs. The size of hetero-nanocomposites of MNPs haphazardly modified onto the bigger Cu_2_O NPs was observed in the submicron range and further used to induce magneto-mechanical stress in *S. cerevisiae* (Fig. 7a(iv)). Further, *S. cerevisiae* cells were reported to be unaffected by the building blocks of NiFe_2_O_4_ NPs (Fig. 7b(i–ii)), their sensitivity to NCs remarkably enhanced on short-time exposure to a rotating low-frequency magnetic field. On careful investigation, it was observed that the fungal membrane showed a mark of disruption, which indicates damage to DNA (Fig. 7b(iii–vii)) [111].

### Zn-based nanoparticles

Meraat et al. [112] synthesized Zn oxide nanoparticles by employing a low temperature sol–gel process annealed at 400 and 550 °C. The synthesized Zinc oxide NPs was characterized by XRD, TEM, and FT-IR. The TEM result showed the hexagonal wurtzite structure of the NPs, with grain sizes ranging between 38 and 43 nm. Farzana et al. [113] synthesized zinc oxide NPs and evaluated their antimicrobial activity against *K. pneumonia* and *E. coli*. This approach could be useful to fabricate nanodrug conjugates as potential agents in different realms of biomedical and pharmaceutical sciences.

Lalabadi et al. [114]*,* suggested the findings revealed that Gram-negative bacteria were more susceptible than antimicrobial agents. The cell wall of Gram-positive bacteria possesses a dense skin of peptidoglycan that has caused more resistance to Gram-negative antimicrobial agent bacteria. Gram-negative bacteria possesses a thin peptidoglycan cap, which enables the movement of metal ion NPs to the cell and also aids in the degree of a lack of thick peptidoglycan coating communicating with the nanoparticles and cell wall of bacteria. The negativity of the lipopolysaccharide layer is also a feature of Gram-negative bacteria [115, 116]. This charge plays a significant role in integrating positive ions, which then contribute to nanoparticles, intracellular damage, and DNA and protein degradation [117].

**Fig. 8:**
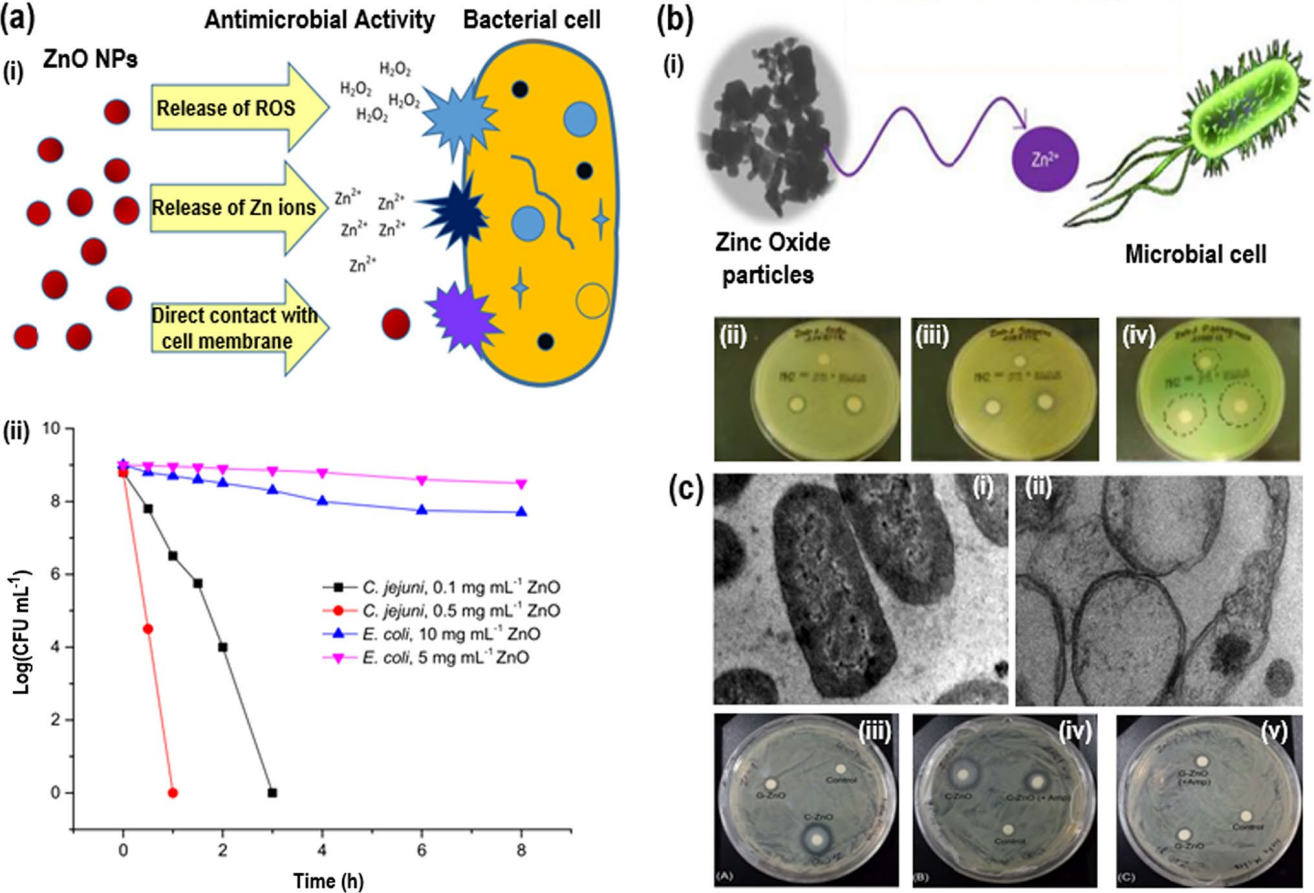
**a** (i) ZnO disinfection mechanisms, (ii) Antibacterial activities of ZnO-NPs against *C. jejuni* and *E. coli* at different ZnO concentrations. Reproduced with permission [118]. Copyright 2021. **b** (i) TEM image of ZnO-NPs and ZnO-NPs action with microbial cell, (ii–iv) Antimicrobial inhibition zones around ZnO impregnated disks. Reproduced with permission [119]. Copyright 2021 Elsevier Publication Group. **c** TEM images of *A. baumannii* cultured in absence (i) and presence (ii) of ZnO-NPs. Disk diffusion assay for ZnO-NPs with *A. baumannii*. Comparative display antibacterial activity of ZnO-NPs prepared via (iii) chemical (C-ZnO) and green (G-ZnO) methods, (iv) chemical (C-ZnO) and ampicillin chemical (C-ZnO + amp) methods, and (v) green (G-ZnO) and ampicillin green (G-ZnO + amp) methods. Reproduced with permission [121] (This is an open-access article distributed under the terms of the Creative Commons Attribution (CC BY) license)

The antimicrobial properties of ZnO-NPs were discussed, indicating the various antimicrobial mechanisms for ZnO-NPs (Fig. 8a(i)). A comparative study showed the disinfection mechanisms for ZnO-NPs, indicating their potential application to water disinfection (Fig. 8a(ii)) [118]. Further, Julia et al. [119] conducted a comprehensive study on medium and dissolution of Zn from ZnO suspensions. For the suspensions of three ZnO grades in broth and the isolated liquid phase comprising soluble Zn species, the antibacterial properties against the five microbes were evaluated. The findings showed that a substantial contribution to the optimal therapeutic potential of ZnO was made by Zn^2+^ generated in the broth (Fig. 8b(i)). The solubility of the Zn in the liquid phase was improved by the complex formation of Zn^2+^ ions by the broth materials. In relation to the bacterial species [120], the corresponding behaviors of the soluble Zn species and ZnO nanoparticles displayed sensitivity. Due to their larger specific area, dissolution was quicker for large levels of ZnO and for ZnO powder. These circumstances have resulted in a stronger antimicrobial potency of ZnO powder (Fig. 8b(i–iv)). ZnO appeared as a valuable substitute for soluble Zn salts, including Zn gluconate. Tiwari et al. [121]*,* synthesized ZnO-NPs and characterized using different spectroscopies. ZnO-NPs prepared are 30 nm in size and have properties corresponding to ZnO-NPs. The TEM images of *A. baumannii* cultured in the absence and presence of chemically synthesized ZnO-NPs are shown in Fig. 8c(i–ii). To investigate the antimicrobial efficacy of ZnO-NPs, the disk diffusion method was employed growth kinetics and disk diffusion studies revealed that ZnO-NPs displayed effective bactericidal performance against carbapenem-resistant *A. baumannii*. Mode of action of ZnO-NPs on the carbapenem-resistant strain of *A. baumannii*. The suggested mechanism of action of ZnO requires the formation of ROS, which increases the lipid peroxidation of the membranes, causing the spillage of sugar, DNA [122], protein, and cellular uptake to the membrane. Such findings have shown that ZnO NP can be established as an alternate therapy for *A. baumannii* (Fig. 8c(iii–v)) [123]. Table 1 showed the advantages and disadvantages of metallic NPs compared to other materials [124, 125].

**Table 1:** Advantages and disadvantages of nanoparticle compared to other materials

Advantages of metallic nanoparticles	Disadvantages of metallic nanoparticles
Delivery of drugs to specified tissues via accumulation	Nanoparticles administered intravenously accumulate in many body organs
Their antimicrobial agents have less adverse effects	Optimal therapeutic effect from locally provided nanoparticle-assisted drugs with high cumulative exposure
Resistance against bacteria is reduced	High systemic absorption of drugs delivered topically with appropriate dosing
Can go across barriers in tissue (such as the blood–brain barrier)	Various organs and systems can be negatively affected by nanotoxicity
Longer duration of therapeutic effect because of elimination delay	Require advanced characterization techniques for the nanoparticles
Modulated release of drugs	
Comprehensive drug index	
Facilitated dissolution	
Reduced immune suppression	

## Conclusion remarks and future prospective

In summary, the extensive investigations have already been conducted with innovative and non-conventional antimicrobial medicines to design and produce suitable medicines capable of addressing drug resistance issues across the globe. NPs in general develop bactericidal activities via ROS capable of interfering metabolic mechanisms, and DNA synthesis, causing cell death. A critical analysis of various types metallic NPs in terms of antimicrobial potential correlated to properties like shape, size, and zeta potential, including metallic poisoning specifically for biomedical applications, has been made to address the issue of drug resistivity and offer a potential alternative against bacterial infections. In addition, multidrug resistance trends are initiating bacteria to develop resistance to many types of antibiotics, and therefore medical professionals considered NPs as a suitable alternative to antibiotics. The antimicrobial potency of NPs was found owing to oxidative or non-oxidative stress, ROS, etc. However, there are still obstacles to implementing NPs in the clinical phase: (i) precise assessment of NPs interactions with cells, tissues, and organs to monitor dose calibration and selection of routes of drug NPs administration, (ii) NPs can be used in an in vivo model, but an in vitro approach is recommended for a more thorough understanding of their toxicity, metabolism, and biocompatibility, (iii) serious problems can arise after intravenous injection or inhalation of NPs because the particles can accumulate in various organs and tissues, and (iv) NPs-treated bacteria displayed a higher generation of ROS compared to untreated bacteria, causing oxidative stress, which can down-regulate the apoptotic gene process. This results in programmed cell death because of leakage in the mitochondrial membrane [126]. In addition, a high ROS level can also promote the possibility of cancer, diabetes, cardiovascular diseases, etc. Therefore, in order to properly understand the use of NPs in bacterial treatment and to take the necessary precautions to monitor such problems, more research is needed. These obstacles must be overcome in order to bring about the cost-effective translation of NPs to the clinic, and this calls for facile and less toxic strategies for NPs fabrication, followed by advanced characterization to demonstrate biocompatibility, nanotoxicology assays, and methods to facilitate easy comparison of data originating from in vitro and in vivo studies.

Moreover, in the context of biomedical hazardous waste management, the public, relevant officials, and the government share responsibility for the appropriate execution and management of biomedical hazardous waste. However, inadequate knowledge and awareness contribute to mishandled biomedical waste. Therefore, there must be a public education and awareness campaign to raise consciousness about the dangers of biomedical waste to human health. An environmentally sustainable, cutting-edge process for treating biomedical hazardous waste is needed. NPs are also crucial in the treatment of biomedical hazardous waste. Many contaminants, such as metals, metalloids, drugs, dyes, pharmaceuticals, and organic pollutants, must be removed from waste water before it can be released into water bodies, and the ability of NPs to remove organic contamination is encouraging. However, NPs being more effective with use of less amount in biomedical as well as antimicrobial treatments, they appear to be the material of choice to impact the future expansion of hazardous waste management. Although this approach is completely successful at laboratory scale, its industrial scale applicability is yet to be ascertained, which will demand a lot of work to find an acceptable solution [127].

## Data Availability

The datasets generated during and/or analyzed during the current study are available from the corresponding author on reasonable request.
